# Characteristics of insulin-Naïve people with type 2 diabetes who successfully respond to insulin glargine U100 after 24 weeks of treatment: a meta-analysis of individual participant data from 3 randomized clinical trials

**DOI:** 10.1186/s40842-018-0059-2

**Published:** 2018-05-08

**Authors:** M. H. Cummings, D. Cao, I. Hadjiyianni, L. L. Ilag, M. H. Tan

**Affiliations:** 1 Queen Alexandra Hospital, Portsmouth, UK; 2 Eli Lilly and Company, Indianapolis, IN USA; 3 Lilly Deutschland GmbH, Bad Homburg, Germany; 4 University of Michigan, Ann Arbor, MI USA

**Keywords:** Type 2 diabetes, Insulin glargine, Insulin naïve, Responders, HbA1c composite endpoint, Baseline characteristics

## Abstract

**Background:**

To identify baseline/clinical characteristics associated with clinically meaningful responses to insulin glargine 100 U/mL (IGlar) in insulin-naive people with type 2 diabetes mellitus (T2DM).

**Methods:**

Individual participant data were pooled from 3 randomized trials to compare baseline characteristics and clinical outcomes associated with 24-week response to IGlar in combination with non-insulin antihyperglycemic agents in participants with T2DM. Responders were defined as achieving endpoint HbA1c target < 53 mmol/mol (< 7%) and/or ≥ 11 mmol/mol (≥ 1%) HbA1c reduction from baseline.

**Results:**

Differences in baseline characteristics for responders versus nonresponders were higher HbA1c (99 vs 91 mmol/mol [9.1 vs 8.3%]; *P* < 0.001), higher fasting blood glucose (FBG; 10.4 vs 8.8 mmol/L [187 vs 159 mg/dL; *P* < 0.001), and fewer participants (94% vs 98%; *P* = 0.006) taking oral medications targeting postprandial blood glucose (BG). Most participants (80%) achieved one or both components of composite endpoint. 12-week response was a strong predictor of subsequent 24-week response (sensitivity, 85.9%; predictive positive value, 91.4%). At both 12 and 24 weeks, < 40% of responders and nonresponders reached target FBG ≤ 5.6 mmol/L (≤ 100 mg/dL). Responders at 24 weeks had higher incidence of hypoglycemia (total, 82.5% vs 70.4%; *P* < 0.001; nocturnal, 60.3% vs 50.5%; *P* = 0.002; documented symptomatic, 65.8% vs 55.6%; *P* < 0.001) than nonresponders.

**Conclusions:**

Baseline characteristics associated with response were identified. The strong predictability of 12-week response suggests that the magnitude of early HbA1c reduction should be considered when assessing response to IGlar. More aggressive IGlar titration may be reasonable for nonresponders and responders who have not reached FBG and HbA1c targets, taking into account other BG timepoints.

**Electronic supplementary material:**

The online version of this article (10.1186/s40842-018-0059-2) contains supplementary material, which is available to authorized users.

## Background

Dual or triple therapy with insulin (usually a basal insulin) in combination with metformin or other noninsulin antihyperglycemic medication is recommended for people with type 2 diabetes mellitus (T2DM) who do not attain glycemic target on noninsulin antihyperglycemic medications alone [1]. For people with newly diagnosed T2DM who are symptomatic and/or have highly elevated levels of glycated hemoglobin (HbA1c) (≥ 86 mmol/mol [≥ 10%]) and/or blood glucose (BG) (≥ 16.7 mmol/L [≥ 300 mg/dL]), basal-bolus insulin (preferred if symptomatic) or basal insulin plus a glucagon-like peptide-1 receptor agonist (GLP1-RA) should be considered for initial treatment [1]. Insulin glargine 100 units/mL (IGlar) was the first basal insulin analogue, which provided almost 24-h glycemic control with once-daily injection and lower risk for nocturnal hypoglycemia compared with human neutral protamine Hagedorn insulin, while having similar efficacy in terms of number of participants reaching HbA1c targets [2–6]. IGlar has since become a benchmark for the development of novel basal insulin analogues [7]. These newer marketed basal insulins, insulin degludec and insulin glargine 300 units/mL, are non-inferior to IGlar in terms of efficacy and in certain subpopulations may provide some advantages over IGlar, such as reduced nocturnal hypoglycemia, as shown in some insulin degludec 100 units/mL studies [8–10] and in insulin glargine 300 units/mL studies of participants already receiving high-dose basal insulin [11, 12]. IGlar, however, remains an important option for the management of T2DM.

In recent years, guidelines and position statements recommend that the choice of pharmacologic agents are to be guided by a patient-centered approach that considers factors like efficacy, hypoglycemia risk, and impact on weight. Even though this method allows for individualization of treatment based on several clinical and social factors, it has become increasingly challenging for healthcare providers to select from a plethora of antihyperglycemic classes and furthermore from different products within each class. Moreover, and specifically for basal insulin, recent literature emphasized the importance of proper titration before treatment intensification [13–15]. Anticipating treatment failures (e.g., minimal improvements, frequent and/or severe hypoglycemia) and establishing realistic expectations of treatment with individuals are important in clinical practice. To reach such decisions, it would be helpful to understand the characteristics of people with T2DM who do or do not respond to a common starter insulin, IGlar.

A few studies have investigated clinical characteristics of people with diabetes on IGlar achieving a target HbA1c of 53 mmol/mol (7%) [13–16]. These studies showed that only a few baseline characteristics were consistently associated with reaching target: lower HbA1c at baseline and shorter duration of diabetes [14, 16]. Furthermore, it was shown that as baseline HbA1c increased, so did mean reduction in HbA1c from baseline; however, progressively fewer participants achieved target HbA1c at study endpoint with every 1% increase in baseline HbA1c. Therefore, these studies were focused mainly on whether patients reached target or not, with no particular attention to patients who may have experienced clinically significant reductions in HbA1c (albeit short of reaching the HbA1c of 7%).

Recent analyses have shown that a composite HbA1c measured by a decrease from baseline in HbA1c of ≥ 11 mmol/mol (≥ 1%) and/or achievement of HbA1c target < 53 mmol/mol (< 7%) identifies more patients with clinically meaningful responses to insulin therapy than attainment of target HbA1c alone [17, 18]. Indeed, it has been shown that a decrease in the HbA1c value of ≥11 mmol/mol (≥1%) may confer clinical benefit, as demonstrated by the UK Prospective Diabetes Study: every 11 mmol/mol (1%) reduction in mean HbA1c level was associated with reductions of 21% for any diabetes-related endpoint, 21% for diabetes-related deaths, 14% for myocardial infarctions, and 37% for microvascular complications [19].

This current post hoc analysis was conducted using an integrated database of prospective clinical studies of once-daily IGlar treatment among insulin-naïve people with T2DM. The aims of this analysis were two-fold: 1) to identify factors/characteristics of responders to once-daily IGlar at 24 weeks, and 2) to assess the sensitivity and specificity of this composite HbA1c response measure at 12 weeks in predicting response at 24 weeks. Response was defined as a composite endpoint: achieving HbA1c < 53 mmol/mol (< 7%) and/or a ≥ 11 mmol/mol (≥ 1%) reduction in HbA1c from baseline. This definition of response has been previously studied [17, 18], and therefore was selected for this analysis based on previous findings and its practical application in the real-world setting.

## Methods

### Integrated database

Individual participant data from 2 randomized clinical trials sponsored by Eli Lilly and Company [20, 21] and 1 randomized trial sponsored by Eli Lilly and Company and Boehringer Ingelheim [22] were used for meta-analyses. These studies were identified by an exhaustive search of Eli Lilly and Company’s integrated clinical trial database based on the following inclusion criteria: 1) participants were insulin-naïve with T2DM, 2) a sufficient number of participants received IGlar (Basaglar®/Abasaglar®, Boehringer Ingelheim and Eli Lilly and Company, or Lantus®, Sanofi-Aventis) for at least 24 weeks, and 3) IGlar was the only insulin component in the antihyperglycemic treatment.

Buse et al. [20] compared the efficacy and safety of twice-daily (BID) insulin lispro mixture 75/25 and once-daily (QD) IGlar in a randomized, open-label, 24-week, non-inferiority trial conducted in 11 countries (Argentina, Australia, Brazil, Canada, Greece, Hungary, India, the Netherlands, Romania, Spain, and the United States). Eligible participants were insulin-naïve adults ≥ 18 years of age with T2DM and taking ≥ 2 oral antihyperglycemic medications (OAMs). Jain et al. [21] compared the efficacy and safety of 2 progressive insulin regimens, QD insulin glargine plus insulin lispro administered up to 3 times daily (TID) versus insulin lispro mixture 50/50 administered up to TID, in a randomized, open-label, 36-week, non-inferiority trial conducted in 9 countries (Australia, Canada, France, Greece, India, Republic of Korea, Mexico, Russian Federation, and Spain). Eligible participants were insulin-naïve adults ≥ 18 years of age with T2DM and taking ≥ 2 OAMs. Rosenstock et al. [22] compared the efficacy and safety of 2 IGlar products, LY IGlar versus Lantus®, in a phase 3, randomized, double-blind, 24-week, non-inferiority trial conducted in 11 countries (Czech Republic, France, Germany, Greece, Hungary, Italy, South Korea, Mexico, Poland, Spain, and the United States). Eligible participants were adults ≥ 18 years of age with T2DM who were either insulin-naïve or previously on Lantus® and taking ≥ 2 OAMs.

In each of these trials [20–22], postprandial glucose (PPG) was collected through 7-Point self-monitored blood glucose (SMBG) at weeks 0, 12, and 24 on 3 separate days in the 2-week period prior to each visit. Participants were also given a diary to record hypoglycemia events experienced throughout each study. For each hypoglycemia episode, the participant was asked to record the glucose value, if measured, and to describe the treatment, including if the participant was able to self-treat, and the outcome of the episode. At the onsite visit, the investigator reviewed the diary with the participant to verify and assess any need for treatment adjustment. Further study design details and key outcomes from each trial are summarized in Additional files 1 and 2, respectively.

### Statistical analysis

Participants with non-missing HbA1c at 24 weeks were analyzed and classified into 2 responder cohorts (yes vs no) according to the composite HbA1c responder measure: HbA1c < 53 mmol/mol (< 7%) at 24 weeks or reduction in HbA1c from baseline to 24 weeks ≥ 11 mmol/mol (≥ 1%). Responders were the participants who either had HbA1c < 53 mmol/mol (< 7%) or had HbA1c reduction ≥ 11 mmol/mol (≥ 1%) at 24 weeks. Nonresponders did not meet either criterion. For the 3 SMBG profiles obtained at 0, 12, and 24 weeks, the average SMBG value was used for analysis. Missing data at week 24 was expected due to the self-monitoring nature of SMBG and the attrition throughout the trials. Overall, about 60% of participants had non-missing PPG data at week 24. More specifically, among the 1485 patients who had non-missing HbA1c values at 24 weeks, 826 participants had non-missing glucose values post-breakfast and post-midday meal and 829 participants had non-missing post-dinner glucose at week 24. This was considered adequate for analysis with the majority of participants contributing data and a total sample size of > 800.

Heterogeneity across the studies was assessed by study-by-responder interaction. *P* values for interaction were nonsignificant for the majority of outcomes measured indicating that results in these trials were relatively homogeneous and therefore justified the integration of these data. In 2 of the 3 studies analysed [20, 22], responders had greater reductions in fasting blood glucose (FBG) than nonresponders, while in the 3rd study [21], the 2 cohorts had similar reductions in FBG, thereby resulting in a statistically significant (*P* = 0.012) study-by-responder interaction (Additional file 2). Baseline participant characteristics and clinical profiles at 24 weeks (HbA1c, FBG, PPG, insulin dose, and hypoglycemia categories [total hypoglycemia (BG ≤ 3.9 mmol/L [≤ 70 mg/dL] or signs/symptoms), documented symptomatic (BG ≤ 3.9 mmol/L [≤ 70 mg/dL] and signs/symptoms), nocturnal (between bedtime and waking), and severe (required 3rd party assistance) were compared between the responder cohorts. A 2-sided *P* value of < 0.05 was considered statistically significant, with *P* values based on the Pearson’s Chi-square test for categorical variables and fixed effects meta-regression model for continuous variables. Results presented are model-adjusted mean and standard error (SE). Relationships between improvements in glycemic outcomes as continuous variables (FBG, daily mean PPG, and HbA1c) and baseline variables (HbA1c, FBG), between improvements in glycemic outcomes as continuous variables (FBG, daily mean PPG, and HbA1c) and insulin dose, and between hypoglycemic rate and insulin dose, were explored graphically using scatter plots as post hoc analyses.

Sensitivity, specificity, positive predictive value, and negative predictive value were evaluated to assess if early response at 12 weeks could predict subsequent response at 24 weeks to support current guidelines that recommend evaluation of therapeutic response to pharmacologic interventions at 12 weeks after initiating therapy.

All analyses were performed using SAS Version 9.2® or higher.

## Results

### Composite versus single HbA1c measure

The majority (80%) of participants achieved a meaningful clinical reduction in HbA1c at 24 weeks, as defined by the composite endpoint of attainment of target HbA1c < 53 mmol/mol (< 7%) and/or a ≥ 11 mmol/mol (≥ 1%) decrease in HbA1c from baseline (Table 1). Of those participants who responded to treatment, 50% achieved both components of the composite HbA1c endpoint, while 43% only experienced a ≥ 11 mmol/mol (≥1%) decrease in HbA1c and 7% only reached target HbA1c < 53 mmol/mol (< 7%). The composite HbA1c endpoint identified 34% more responders than would have been found by the single HbA1c endpoint, achievement of target HbA1c < 53 mmol/mol (< 7%), commonly used in diabetes clinical studies.

**Table 1:** Number of responders and nonresponders to insulin glargine 100 Units/mL at 24 weeks by composite HbA1c endpoint

HbA1c < 7% (Yes/No)	≥1% reduction in HbA1c (Yes/No)	Responders (HbA1c < 7% or ≥ 1% reduction) n (%)	Nonresponders (HbA1c ≥ 7% and < 1% reduction) n (%)
Yes	Yes	595 (50)	–
Yes	No	88 (7)	–
No	Yes	505 (43)	–
No	No		297 (20)
Total (*N* = 1485)		1188 (80)	297 (20)

*Abbreviation*: *HbA1c* glycated hemoglobin

### Baseline characteristics of responders and nonresponders

The baseline characteristics of responders (*N* = 1188) and nonresponders (*N* = 297) at 24 weeks were generally similar; however, there were some notable differences (Table 2). More men than women (54 vs 46%; *P* = 0.012) responded to treatment. Responders also had higher baseline HbA1c levels (mean, 9.1 vs 8.3% [99 vs 91 mmol/mol]; *P* < 0.001), higher FBG levels (10.4 vs 8.8 mmol/L [187 vs 159 mg/dL; *P* < 0.001), and had fewer participants (94 vs 98%; *P* = 0.006) who used OAMs targeting PPG than nonresponders. Overall, both responders and nonresponders at 24 weeks had a baseline mean duration of T2DM of 10 years and were generally Caucasian (71 vs 58%), less than 65 years of age (75 vs 76%), and overweight or obese (mean body mass index [BMI] for both, 31 kg/m^2^) at baseline.

**Table 2:** Baseline characteristics of responders and nonresponders to insulin glargine 100 Units/mL at 24 weeks

	Responders at 24 weeks (HbA1c < 7% or ≥ 1% reduction) *n* = 1188	Nonresponders at 24 Weeks (HbA1c ≥ 7% and < 1% reduction) *n* = 297	*P* value
Age, years	57.8 (9.9)	57.2 (10.1)	0.323
Age group			0.833
< 65 years	893 (75.2)	225 (75.8)	
≥ 65 years	295 (24.8)	72 (24.2)	
Gender			0.012
Women	543 (45.7)	160 (53.9)	
Men	645 (54.3)	137 (46.1)	
Duration of T2DM, years	10.3 (6.6)	9.7 (6.5)	0.159
Weight, kg	87.5 (19.7)	85.8 (21.6)	0.201
BMI, kg/m^2^	31.3 (5.70)	30.9 (5.70)	0.213
HbA1c, %	9.1 (1.2)	8.3 (0.9)	< 0.001
HbA1c, mmol/mol	99 (13)	91 (10)	< 0.001
FBG, mg/dL	186.7 (52.0)	158.5 (42.2)	< 0.001
≥ 1 OAMs Targeting PPG^a^	1111 (93.5)	290 (97.6)	0.006
SU, yes	1067 (89.8)	284 (95.6)	0.002
2 OAMs	790 (66.5)	194 (65.3)	0.701
MET/SU	614 (51.7)	167 (56.2)	–
3 OAMs	389 (32.7)	100 (33.7)	0.761
MET/SU/TZD	346 (29.1)	89 (30.0)	**–**
4 OAMs	7 (0.6)	3 (1.0)	0.429

Individual participant data were pooled from 3 randomized clinical trials [20–22] and are mean (SD) or n (%). Patients are those who were randomized to insulin glargine as the only insulin treatment and with no missing HbA1c values at 24 weeks. Two-sided *P* values were considered statistically significant if < 0.05 and were calculated by ANOVA model (response = subgroup) for continuous variables and by Pearson’s Chi-square or Fisher’s exact (for OAM data with less than 80% of cells with an expected value ≥5) test for categorical variables. *Abbreviations*: *AGI* alpha glucosidase inhibitor, *BMI* body mass index, *DPP-IV* dipeptidyl peptidase IV, *FBG* fasting blood glucose, *HbA1c* glycated hemoglobin, *MEG* meglitinides, *MET* metformin, *OAM* oral antihyperglycemic medication, *PPG* postprandial blood glucose, *SD* standard deviation, *SU* sulphonylurea, *T2DM* type 2 diabetes mellitus, *TZD* thiazolidinedione. ^a^OAMs targeting postprandial glucose were SU, DPP-IV, AGI, and MEG

### Glycemic response and insulin dose

**Fig. 1:**
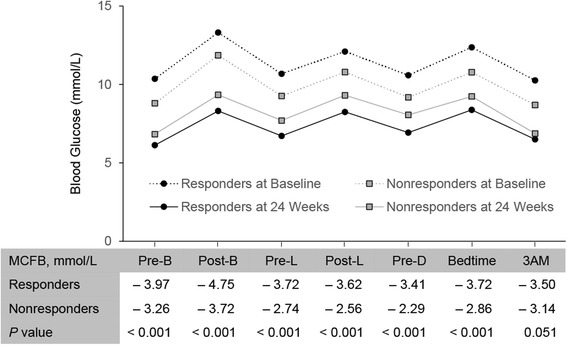
Blood glucose over time in responders and nonresponders to insulin glargine 100 Units/mL at 24 Weeks. Blood glucose values for SMBG time points are daily mean. *Abbreviations*: *MCFB* mean change from baseline, *Pre-B* before breakfast, *Pre-L* before lunch or midday meal, *Pre-D* before dinner, *Post-B* 2 h after breakfast, *Post-L* 2 h after lunch or midday meal

**Fig. 2:**
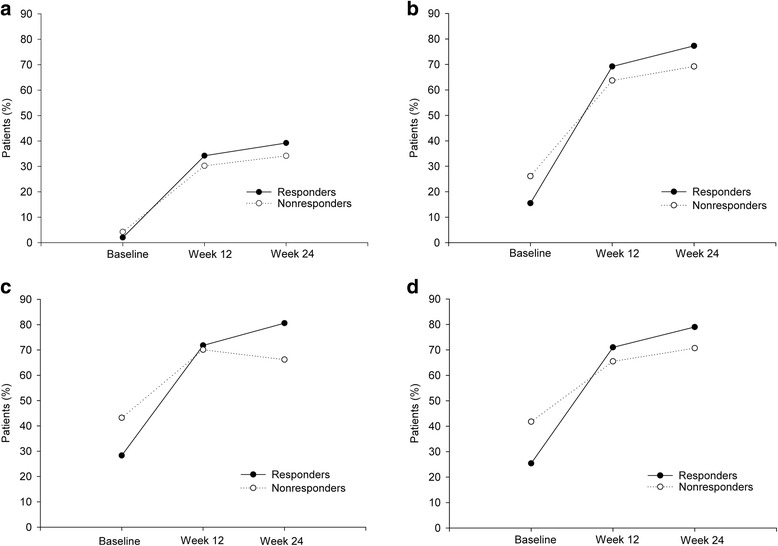
Responders and nonresponders to insulin glargine 100 Units/mL at 24 weeks who reached blood glucose targets. Data are percentage of responders and nonresponders who reached target fasting blood glucose (≤5.6 mmol/L; ≤100 mg/dL) (**a**) and target postprandial glucose (≤ 180 mg/dL) after breakfast (**b**), lunch (**c**), and dinner (**d**). The postprandial glucose target was defined for this analysis and was not given to investigators during these trials

Early response at 12 weeks was a strong predictor of subsequent response at 24 weeks as shown by the high sensitivity (85.9%) and predictive positive value (91.4%) (Table 3). Responders at 24 weeks had significantly greater reductions from baseline in adjusted mean (SE) HbA1c (− 2.2% [0.04] vs − 0.8% [0.06]; − 24 [0.44] vs − 9 [0.66] mmol/mol; *P* < 0.001) than nonresponders (Table 4), as to be expected per the definition of response. Responders compared with nonresponders also had significantly greater reductions from baseline in both adjusted mean (SE) daily FBG (− 4.0 [0.09] vs − 3.3 [0.16] mmol/L; − 71 [1.6] vs − 59 [2.8] mg/dL; *P* < 0.001) and daily PPG (− 4.1 [0.11] vs − 3.0 [0.18] mmol/L; − 73 [1.9] vs − 54 [3.3] mg/dL; *P* < 0.001) (Table 4). The difference between responders and nonresponders in change from baseline adjusted mean daily BG levels at 24 weeks was small at pre-breakfast and 3 AM, but more pronounced at other time points (Fig. 1). More responders than nonresponders (39% vs 34%) reached FBG target ≤ 5.6 mmol/L (≤ 100 mg/dL) at 24 weeks (Fig. 2). Responders were also more likely to reach PPG target ≤ 10.0 mmol/L (≤ 180 mg/dL) after breakfast (77% vs 69%), lunch (81% vs 66%), and evening meal (79% vs 71%) than nonresponders (Fig. 2). (Note: the PPG target was defined for this analysis and was not given to investigators during these trials.) The adjusted mean (SE) daily IGlar dose was similar between responders and nonresponders at 24 weeks (45.2 [1.4] vs 42.2 [2.3] units/day; 0.49 [0.01] vs 0.47 [0.02] units/kg/day) (Table 4). From a post hoc graphical assessment (data not shown), inverse linear relationships were observed between improvements in glycemic measures (FBG, daily mean PPG, HbA1c) and baseline HbA1c or FBG which confirm the findings mentioned above. Plots of improvements in glycemic measures and insulin dose (data not shown) did not reveal an informative pattern.

**Table 3:** Prediction of insulin glargine 100 Units/mL response at 24 weeks based on early response at 12 weeks

		Responders at week 24	
		Yes	No
Responders at Week 12	Yes	91%	9%
	No	46%	54%
Predictive parameters			
Odds ratio = 12.7 (*P* < 0.001)			
Sensitivity = 85.9%			
Specificity = 67.7%			
Positive predictive value = 91.4%			
Negative predictive value = 54.5%			

Sensitivity is the percentage of subsequent responders (HbA1c <7% or ≥ 1% reduction) correctly identified (true-positive rate). Specificity is the percentage of subsequent nonresponders (HbA1c ≥7% and < 1% reduction) correctly identified (true-negative rate). Positive predictive value is the percentage of subsequent responders among early responders. Negative predictive value is the percentage of subsequent nonresponders among early nonresponders. *Abbreviation*: *HbA1c* glycated hemoglobin

**Table 4:** Glycemic response and insulin dose in responders and nonresponders to insulin glargine 100 Units/mL at 24 weeks

	Responders (HbA1c < 7% or ≥ 1% reduction)	Nonresponders (HbA1c ≥ 7% and < 1% reduction)
HbA1c (mmol/mol), n	1188	297
Endpoint	74 (0.44)	89 (0.66)
CFB	− 24 (0.44)	− 9 (0.66)
CFB, LSM Diff (95% CI)	− 15 (− 16.40, − 13.88); *P* < 0.001	
HbA1c (%), n	1188	297
Endpoint	6.76 (0.04)	8.14 (0.06)
CFB	− 2.16 (0.04)	− 0.78 (0.06)
CFB, LSM Diff (95% CI)	− 1.38 (− 1.50, − 1.27); *P* < 0.001	
Dose (units/day), n	1187	297
Endpoint	45.21 (1.36)	42.16 (2.29)
LSM Diff (95% CI)	3.05 (− 1.24, 7.34); *P* = 0.164	
Dose (units/kg/day), n	1187	297
Endpoint	0.49 (0.01)	0.47 (0.02)
LSM Diff (95% CI)	0.02 (− 0.02, 0.07); *P* = 0.272	
Fasting blood glucose, n	692	143
Endpoint (mmol/L)	6.13 (0.09)	6.83 (0.16)
CFB	− 3.97 (0.09)	− 3.26 (0.16)
CFB, LSM Diff (95% CI)	− 0.71 (− 1.00, − 0.41); *P* < 0.001	
Endpoint (mg/dL)	110.39 (1.63)	123.10 (2.81)
CFB	− 71.48 (1.63)	− 58.77 (2.81)
CFB, LSM Diff (95% CI)	−12.71 (− 18.00, − 7.42); *P* < 0.001	
Daily mean SMBG, n	640	128
Endpoint (mmol/L)	7.30 (0.09)	8.20 (0.16)
CFB	− 3.85 (0.09)	− 2.95 (0.16)
CFB, LSM Diff (95% CI)	− 0.90 (− 1.20, − 0.60); *P* < 0.001	
Endpoint (mg/dL)	131.45 (1.62)	147.70 (2.83)
CFB	− 69.33 (1.62)	− 53.08 (2.83)
CFB, LSM Diff (95% CI)	− 16.25 (− 21.60, − 10.89); *P* < 0.001	
Daily mean premeal SMBG, n	678	142
Endpoint (mmol/L)	6.59 (0.08)	7.51 (0.14)
CFB	− 3.70 (0.08)	− 2.78 (0.14)
CFB, LSM Diff (95% CI)	− 0.92 (− 1.19, − 0.65); *P* < 0.001	
Endpoint (mg/dL)	118.75 (1.52)	135.30 (2.58)
CFB	− 66.64 (1.52)	− 50.09 (2.58)
CFB, LSM Diff (95% CI)	− 16.55 (− 21.40, − 11.69); *P* < 0.001	
Daily mean postprandial SMBG, n	677	139
Endpoint (mmol/L)	8.31 (0.11)	9.32 (0.18)
CFB	− 4.03 (0.11)	− 3.02 (0.18)
CFB, LSM Diff (95% CI)	− 1.01 (− 1.36, − 0.66); *P* < 0.001	
Endpoint (mg/dL)	149.70 (1.93)	167.91 (3.33)
CFB	− 72.70 (1.93)	− 54.49 (3.33)
CFB, LSM Diff (95% CI)	− 18.21 (− 24.50, − 11.93); *P* < 0.001	
Intrapatient between-day SMBG variability, n	673	137
Endpoint (mmol/L)	0.62 (0.03)	0.71 (0.05)
CFB	− 0.27 (0.03)	− 0.18 (0.05)
CFB, LSM Diff (95% CI)	− 0.08 (− 0.18, 0.01); *P* = 0.070	
Endpoint (mg/dL)	11.20 (0.52)	12.74 (0.89)
CFB	− 4.86 (0.52)	− 3.33 (0.89)
CFB, LSM Diff (95% CI)	− 1.53 (− 3.19, 0.13); *P* = 0.070	

Endpoints and CFB values are expressed as LSM (SE) unless otherwise stated. Pearson’s Chi-square test was used for categorical variables and ANCOVA model (response = baseline of the response variable + responder + study + study-by-responder interaction + sulphonylurea use [yes/no]) for continuous variables. *P* values are based on fixed effects meta-regression with a 2-sided α-level of 0.05. Heterogeneity was assessed by study-by-responder interaction. *P* values for interaction were nonsignificant (≥ 0.05) for outcomes presented with the exception of fasting blood glucose (*P* = 0.012), indicating results in these trials were relatively homogeneous. All patients had HbA1c values at 24 weeks and received insulin glargine as the only insulin therapy. *Abbreviations*: *CFB* change from baseline, *CI* confidence interval, *Diff* difference, *HbA1c* glycated hemoglobin, *LSM* least squares mean, *SE* standard error, *SMBG* self-monitored blood glucose, *T2DM* type 2 diabetes mellitus

### Hypoglycemia

**Fig. 3:**
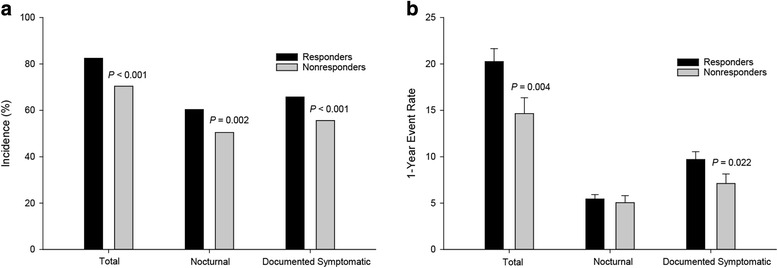
Hypoglycemia in responders and nonresponders to insulin glargine 100 Units/mL at 24 weeks. Data are incidence (**a**) and 1-year event rates (**b**)

At 24 weeks, responders compared with nonresponders had a significantly higher incidence of total (82.5 vs 70.4%; *P* < 0.001), nocturnal (60.3 vs 50.5%; *P* = 0.002) and documented symptomatic (65.8 vs 55.6%; *P* < 0.001) hypoglycemia and significantly higher adjusted mean 1-year event rates of total (20.3 vs 14.7; *P* = 0.004) and documented symptomatic (9.7 vs 7.1; *P* = 0.022) hypoglycemia (Fig. 3). Nocturnal hypoglycemia adjusted mean 1-year event rates (5.4 vs 5.0; *P* = 0.608) and severe hypoglycemia incidence (12 [1.0%] vs 0; *P* = 0.082) and adjusted mean 1-year event rates (0 for both; *P* = 1.000) were similar between groups. From a post hoc graphical assessment (data not shown), no meaningful pattern between hypoglycemia rate and insulin dose was revealed.

## Discussion

This analysis showed that the composite HbA1c response at 12 weeks was a strong predictor of maintaining at least the same HbA1c response (composite) at 24 weeks. Responders had higher HbA1c and FBG levels at baseline with fewer participants using OAMs targeting PPG than nonresponders. Responders compared with nonresponders were also more likely to reach target FBG ≤ 5.6 mmol/L (≤ 100 mg/dL) and *hypothetical* target PPG ≤ 10.0 mmol/L (≤ 180 mg/dL) at 24 weeks with similar IGlar doses. Moreover, adjusted mean IGlar doses at 24 weeks for both groups were still below the limit for basal insulin titration (> 0.5 units/kg/day) recommended by the American Diabetes Association and European Association for the Study of Diabetes [1]. Despite the relatively high averages for IGlar dose and hypoglycemia incidence and yearly event rates at 24 weeks, hypoglycemia incidence and yearly event rates were lower in nonresponders than responders, and both groups had similar yearly event rates of nocturnal hypoglycemia. Given that hypoglycemia data were collected systematically, the chance for under-estimation of hypoglycemia incidence is expected to be small. The high incidence of hypoglycemia in both groups may be explained in part by the aggressive dose-titration protocols used in these trials (Additional file 1), and therefore inadequate titration is unlikely to have caused a lower incidence of hypoglycemia in nonresponders. The mean IGlar dose was close to 0.5 units/kg. Scatter plots of HbA1c versus dose and hypoglycemia yearly event rate versus dose at 24 weeks did not show an informative pattern to suggest a relationship between dose and improvement in glycemic control or lower hypoglycemia incidence. Some people with T2DM, however, may need greater than 0.5 units/kg/day of basal insulin in case of higher insulin resistance. The first-up-titrate-basal insulin approach is expected to be useful in some patients and should be considered on an individual basis. Early introduction of a medication for the control of PPG to reduce the risk of hypoglycemia, especially if using a GLP1-RA, could also benefit some individuals. For nonresponders to IGlar who have reached FBG target or whose up-titration of IGlar dose is limited by the frequency and/or severity of hypoglycemia, diabetes treatment should be intensified with PPG-lowering agents, such as prandial insulin or GLP1-RA.

Our findings are consistent with several post hoc analyses of trials assessing differences between responders and nonresponders to basal insulin treatment. Scheen et al. [13] evaluated the relative contributions of FBG and PPG to overall hyperglycemia in insulin-naive participants who either reached or did not reach target HbA1c < 53 mmol/mol (< 7%) with IGlar or insulin lispro mix 25 at 24 weeks in the DURABLE trial. Insulin doses were higher but hypoglycemia yearly event rates were lower in nonresponders compared with responders at study endpoint. Failure to reach target FBG ≤ 5.6 mmol/L (≤ 100 mg/dL) was the primary reason for not achieving target HbA1c in both insulin groups, suggesting the need to first up titrate basal insulin before intensifying therapy with PPG-lowering agents. Similarly, Khunti et al. [14] found that the suboptimally controlled group (HbA1c ≥ 53 mmol/mol [7%]) treated with once-daily insulin detemir in the SOLVE trial had a relatively low risk of hypoglycemia and suboptimal FBG levels at 24 weeks, which according to the authors suggested that ‘a more aggressive titration regimen could be implemented to improve glycemic control’. A Spanish cross-sectional study looking at people with T2DM on basal insulin also showed that approximately half of the participants had high FBG and HbA1c levels, and hence for those participants, further adjustments of basal insulin would be necessary [15]. Similar patterns of response to basal insulin were observed in a recent meta-analysis of participant-level data from 3415 insulin-naïve participants treated with IGlar in 16 randomized, treat-to-target trials [23]. Participants who reached target HbA1c < 53 mmol/mol (< 7%) at 24 weeks were more likely to achieve target FBG ≤ 5.5 mmol/L (≤ 100 mg/dL), as well as had greater likelihood of reaching target FBG levels without hypoglycemia. Nevertheless, responders had more hypoglycemia events than participants with HbA1c levels of 53 mmol/mol (7%) to 64 mmol/mol (8%) or > 64 mmol/mol (8%). The higher HbA1c groups also had more weight gain and a slightly greater insulin dose at 24 weeks. Interestingly, more frequent hypoglycemia was associated with lower baseline C-peptide levels and was associated with more weight gain at 24 weeks in all HbA1c groups. The authors concluded that people initiating therapy with IGlar are a heterogeneous group in terms of glycemic response and hypoglycemia risk. Our analysis focused on glycemic response, and therefore did not include weight changes from baseline. Baseline C-peptide values were not available to assess.

Studies analyzing the predictability of early response to IGlar for subsequent longer-term response are limited. The high predictive values of 12-week response for subsequent treatment success at 24 weeks in our meta-analysis are consistent with results of a pooled analysis of participant-level data by Fu et al. [17] using the same composite HbA1c endpoint to measure response to IGlar and to OAMs in 3 randomized clinical trials. The high predictive values of 12-week response in both analyses support current guidelines that recommend clinicians evaluate therapeutic responses to pharmacologic interventions 12 weeks after initiating therapy [1].

Participants with higher baseline HbA1c levels have been shown to be less likely to achieve target HbA1c < 53 mmol/mol (< 7%) with basal insulin therapy in randomized clinical trials [13, 16] and in observational studies [14]. Other baseline factors associated with not achieving this HbA1c target include longer duration of diabetes [14, 16], use of a sulphonylurea [16], number of OAMs and longer duration of OAM treatment [14], and higher BMI [14]. When achievement of HbA1c < 53 mmol/mol (< 7%) was further analysed by reaching target FBG < 7.2 mmol/L (< 130 mg/dL) in an observational cross-sectional study of 9899 participants with T2DM [15], only 18% of the study population achieved both HbA1c and FBG targets. Of the participants who did not reach HbA1c target (75% of the total population), those who reached target FBG (24%) were older, had a longer duration of diabetes, and had lower mean values for HbA1c, BMI, diastolic blood pressure, and low-density lipoprotein cholesterol at baseline than those who did not reach target FBG (51%).

Limitations of this meta-analysis are in part due to its post hoc design, and therefore a cause and effect relationship cannot be established. Additionally, data are from randomized clinical trials, thereby results may not reflect what would be seen in a real-world setting, where more aggressive titration of basal insulin is possible and the addition of prandial insulin or a GLP1-RA is an option. Additionally, a lesser reduction in HbA1c (e.g., 8.7 mmol/mol [0.8%] vs 11 mmol/mol [1%]) despite not reaching target HbA1c < 53 mmol/mol [< 7%] could be considered clinically significant. Reasons for not increasing IGlar dose to reach HbA1c goal, particularly for participants without hypoglycemia, were not assessed in these trials, and insulin resistance was not measured. Treatment algorithms and noninsulin treatment options have also changed since these studies were conducted, and therefore results of our analysis may not fully reflect today’s clinical practice.

## Conclusions

As would be expected, more participants with clinically meaningful reductions in HbA1c were identified in this analysis by expanding the definition of response to include a ≥ 11 mmol/mol (≥ 1%) reduction in HbA1c from baseline. Baseline HbA1c and FBG were also statistically higher in responders than nonresponders, although there was not a significant clinical difference to warrant a cut-off for basal insulin administration. At both 12 and 24 weeks, the majority of responders and nonresponders had not reached target FBG, suggesting that more aggressive insulin titration was still possible in responders who had not yet reached FBG and HbA1c goals, assuming hyper- or hypoglycemia at other self-monitoring BG time points were not the driving factors. These findings, coupled with the high predictability of 12-week response for subsequent longer-term response, suggest that the composite HbA1c endpoint is superior to the single HbA1c measure. Clinicians should consider the magnitude of reduction in HbA1c at 12 weeks in addition to achievement of target HbA1c to better recognize the potential for success with this effective starter basal insulin, especially for people with more advanced T2DM and/or higher HbA1c levels, which may require larger insulin doses to obtain glycemic control possibly related to higher insulin resistance. Moreover, sequentially targeting FBG and PPG levels could identify nonresponders to IGlar who could become responders with additional therapy beyond basal insulin. This approach could be encouraging to people with T2DM who are clinically benefiting early from IGlar therapy to continue treatment to get closer to desired goal. A better understanding of concerns of clinicians and/or people with T2DM and reasons for not up titrating or intensifying IGlar therapy is needed and is currently being assessed in a large observational study [24–26]. Studies evaluating the value of adding basal-only versus basal-bolus insulin or GLP1-RA treatment in people with T2DM not adequately controlled on current noninsulin combination therapies are needed to inform clinicians’ treatment selection.

Our findings confirm the clinical utility of IGlar as a safe and effective starter basal insulin and offer insights about addressing an individual’s response to treatment as early as 12 weeks. Timely adjustments to basal insulin therapy could help keep people with T2DM on track to benefit from better glycemic control.

## Additional files

Additional file 1: **Table S1.** Summary of trials integrated for meta-analysis. Overview of the design of each trial used in meta-analysis. (DOCX 34 kb)

Additional file 2: **Table S2.** Outcomes of responders and nonresponders to IGlar at 24 weeks by study. Data are HbA1c, insulin dose, and FBG change from baseline to 24 weeks categorized by response (i.e., responders and nonresponders) in each study. (DOCX 35 kb)

## Abbreviations

BG: Blood glucose
BMI: Body mass index
FBG: Fasting blood glucose
GLP1-RA: Glucagon-like peptide-1 receptor agonist
HbA1c: Glycated hemoglobin
IGlar: Insulin glargine 100 units/mL
OAM: Oral antihyperglycemic medication
PPG: Postprandial blood glucose
SE: Standard error
SMBG: Self-monitored blood glucose
T2DM: Type 2 diabetes mellitus
UK: United Kingdom
