# Supplementary material for: Characteristics of insulin-Naïve people with type 2 diabetes who successfully respond to insulin glargine U100 after 24 weeks of treatment: a meta-analysis of individual participant data from 3 randomized clinical trials
Source: Clin Diabetes Endocrinol. 2018 May 8;4:10. doi: 10.1186/s40842-018-0059-2 (PMC5941643; doi:10.1186/s40842-018-0059-2)
Supplement: Supplementary file 1 — Table S1. Summary of trials integrated for meta-analysis. Overview of the design of each trial used in meta-analysis. (DOCX 34 kb) [file 40842_2018_59_MOESM1_ESM.docx]

**Additional file 1: Table S1** Summary of trials integrated for post hoc analysis

| **Study** | **Design** | **Treatments** | **Patients** | **Primary Endpoint** | **HbA1c Targets** | **Insulin Glargine Titration** |
| --- | --- | --- | --- | --- | --- | --- |
| Buse *et al*.  2009 [20] | efficacy/safety  randomized  parallel  open-label  multinational  24-week duration | BID LM 75/25 (N=1045) vs.  QD IGlar (N=1046) | T2DM  Insulin-naïve  Aged 30-80 years  HbA1c >53 mmol/mol (>7%)  ≥2 OAMs (MET, SU, TZD) | Change in HbA1c from baseline to 24-week endpoint | ≤48 mmol/mol (≤6.5%)  <53 mmol/mol (<7%) | Physician-driven titration: dose decrease by ‑2 units for FBG <4.4 mmol/L (<80 mg/dL), no change for FBG 4.4-5.5 mmol/L (80-100 mg/dL), or dose increase by +2, +4, +6, or +8 units for FBG 5.6-6.7 mmol/L (101-120 mg/dL), 6.8-7.8 mmol/L (121-140 mg/dL), 7.9-8.9 mmol/L (141-160 mg/dL), or >8.9 mmol/L (>160 mg/dL), respectively. Hypoglycaemia was prioritized over hyperglycaemia. |
| Jain *et al*.  2010 [21] | efficacy/safety  randomized  parallel  open-label  active-control  multinational  36-week duration | Progression from QD to BID or TID LM 50/50 (N=242) vs.  Progression from QD IGlar to QD IGlar + prandial Lispro QD to TID, if needed (N=242) | T2DM  Insulin-naïve  Aged 30-80 years  HbA1c >58-108 mmol/mol (>7.5-12.0%)  ≥2 OAMs (MET, SU, TZD) | Change in HbA1c from baseline to 36-week endpoint | ≤48 mmol/mol (≤6.5%)  <53 mmol/mol (<7%)  ≤53 mmol/mol (≤7%) | Physician-driven titration: dose decrease by ‑2 units for FBG <4.4 mmol/L (<80 mg/dL), no change for FBG 4.5-5.5 mmol/L (81-100 mg/dL), or dose increase by +2, +4, +6, or +8 units for FBG 5.6-6.6 mmol/L (101-120 mg/dL), 6.7-7.7 mmol/L (121-140 mg/dL), 7.8-8.8 mmol/L (141-160 mg/dL), or ≥8.9 mmol/L (≥161 mg/dL), respectively. Hypoglycaemia was prioritized over hyperglycaemia. |
| Rosenstock *et al*. 2015 [22] | efficacy/safety  randomized  parallel  double-blind  active-control  multinational  24-week duration | QD LY IGlar (N=376) vs.  QD IGlar (N=380) | T2DM  Insulin-naïve or previously on IGlar  Aged ≥18 years  HbA1c if insulin-naïve: ≥53 and ≤97 mmol/mol (≥7.0 and ≤11.0%)  HbA1c if previously on IGlar: ≤97 mmol/mol (≤11.0%)  ≥2 OAMs (most common combinations: MET + SU [62.4%] and MET + DDP-4 [10.1%]) | Change in HbA1c from baseline to 24-week endpoint | ≤48 mmol/mol (≤6.5%)  <53 mmol/mol (<7%) | Patient-driven titration: addition of 1 unit daily until FBG levels reached ≤5.6 mmol/L (≤100 mg/dL). |
| BID, twice-daily; DPP-4, dipeptidyl peptidase-4 inhibitors; FBG, fasting blood glucose; HbA1c, glycated haemoglobin; IGlar, insulin glargine 100 units/ml (Lantus®), Lispro, insulin lispro; LM, insulin lispro mixture; LY IGlar, LY2963016 insulin glargine 100 units/ml (Basaglar^®^/Abasaglar^®^); MET, metformin; N, number of randomized patients; OAM, oral antihyperglycaemic medication; QD, once-daily; SU, sulphonylurea; T2DM, type 2 diabetes mellitus; TID, three-times-daily; TZD, thiazolidinedione; vs, versus. | | | | | | |
