# Supplementary material for: Characteristics of insulin-Naïve people with type 2 diabetes who successfully respond to insulin glargine U100 after 24 weeks of treatment: a meta-analysis of individual participant data from 3 randomized clinical trials
Source: Clin Diabetes Endocrinol. 2018 May 8;4:10. doi: 10.1186/s40842-018-0059-2 (PMC5941643; doi:10.1186/s40842-018-0059-2)
Supplement: Supplementary file 2 — Table S2. Outcomes of responders and nonresponders to IGlar at 24 weeks by study. Data are HbA1c, insulin dose, and FBG change from baseline to 24 weeks categorized by response (i.e., responders and nonresponders) in each study. (DOCX 35 kb) [file 40842_2018_59_MOESM2_ESM.docx]

**Additional file 2: Table S2** Key outcomes of responders and nonresponders to insulin glargine 100 units/ml at 24 weeks by study

|  | **Responders**  **(HbA1c <7% or ≥1% Reduction)** | **Nonresponders**  **(HbA1c ≥7% and <1% Reduction)** | ***P*value** |
| --- | --- | --- | --- |
| **HbA1c (mmol/mol), n; LS mean CFB (SE) at 24 weeks** | | |  |
| Buse *et al.* [20] | 677; -24 (0.55) | 193; -7 (0.77) | <0.001 |
| Jain *et al.* [21] | 184; -25 (0.55) | 33; -13 (1.42) | <0.001 |
| Rosenstock *et al.* [22] | 327; -21 (0.44) | 71; -7 (0.87) | <0.001 |
| Integrated analysis | 1188; -24 (0.44) | 297; -9 (0.66) | <0.001 |
| **HbA1c (%), n; LS mean CFB (SE) at 24 weeks** | | |  |
| Buse et al. [20] | 677; -2.16 (0.05) | 193; -0.64 (0.07) | <0.001 |
| Jain et al. [21] | 184; -2.33 (0.05) | 33; -1.15 (0.13) | <0.001 |
| Rosenstock et al. [22] | 327; -1.90 (0.04) | 71; -0.64 (0.08) | <0.001 |
| Integrated analysis | 1188; -2.16 (0.04) | 297; -0.78 (0.06) | <0.001 |
| **Dose (units/kg/day), n; LS mean (SE) at 24 weeks** | | |  |
| Buse *et al.* [20] | 676; 0.43 (0.01) | 193; 0.46 (0.02) | 0.100 |
| Jain *et al.* [21] | 184; 0.45 (0.02) | 33; 0.37 (0.05) | 0.105 |
| Rosenstock *et al.* [22] | 327; 0.57 (0.03) | 71; 0.55 (0.05) | 0.659 |
| Integrated analysis | 1187; 0.49 (0.01) | 297; 0.47 (0.02) | 0.679 |
| **FBG (mmol/L), n; LS mean CFB (SE) at 24 weeks** | | |  |
| Buse *et al.* [20] | 190; -4.55 (0.18) | 43; -3.69 (0.31) | 0.004 |
| Jain *et al.* [21] | 182; -3.86 (0.12) | 30; -3.73 (0.29) | 0.679 |
| Rosenstock *et al.* [22] | 320; -3.72 (0.10) | 70; -2.56 (0.18) | <0.001 |
| Integrated analysis | 692; -3.97 (0.09) | 143; -3.26 (0.16) | <0.001^a^ |
| **FBG (mg/dL), n; LS mean CFB (SE) at 24 weeks** | | |  |
| Buse *et al.* [20] | 190; -81.98 (3.24) | 43; -66.56 (5.58) | 0.004 |
| Jain *et al.* [21] | 182; -69.50 (2.12) | 30; -67.13 (5.28) | 0.679 |
| Rosenstock *et al.* [22] | 320; -67.01 (1.72) | 70; -46.16 (3.19) | <0.001 |
| Integrated analysis | 692; -71.48 (1.63) | 143; -58.77 (2.81) | <0.001^a^ |
| Results of the integrated analysis were calculated by ANCOVA model (response = baseline of the response variable + responder + study + study-by-responder interaction + sulphonylurea use [yes/no]). *P* values presented for the integrated analysis are the difference between responders and nonresponders and are based on fixed effects meta-regression with a 2-sided α-level <0.05. Heterogeneity for the integrated analysis was assessed by study-by-responder interaction. *P* values for interaction were nonsignificant (≥0.05) for the majority of outcomes measured indicating results in these trials were relatively homogeneous. All participants had type 2 diabetes mellitus and were insulin naïve prior to study drug treatment, received insulin glargine as the only insulin therapy, and had no missing HbA1c values at 24 weeks. Abbreviations: CFB, change from baseline; FBG, fasting blood glucose; HbA1c, glycated haemoglobin; LS, least squares; SE, standard error. ^a^FBG *P* value for heterogeneity =0.012. | | | |
